# Integrated inflammation-immune-matrix remodeling biomarkers for diagnosis and stage stratification of primary laryngeal cancer

**DOI:** 10.5937/jomb0-63714

**Published:** 2026-05-22

**Authors:** Kaisheng Lin, Jiongcong Yuan, Ying Wang

**Affiliations:** 1 Jieyang People's Hospital, Department of Otolaryngology, Jieyang, China

**Keywords:** laryngeal cancer, YKL-40, suPAR, MMP-9, CRP/albumin ratio, neutrophil-to-lymphocyte ratio, rak grkljana, YKL-40, suPAR, MMP-9, odnos CRP/albumin, odnos neutrofila i limfocita

## Abstract

**Background:**

Aryngeal squamous cell carcinoma (LSCC) is characterized by complex interactions among systemic inflammation, immune dysregulation, and extracellular matrix remodeling. Circulating biomarkers reflecting these biological processes may provide valuable tools for disease detection and stratification; however, their integrated diagnostic value in primary LSCC remains insufficiently defined.

**Methods:**

We conducted a case-control study including 50 treatment-naïve LSCC patients and 50 healthy controls. Levels of YKL-40, soluble urokinase plasminogen activator receptor (suPAR) and matrix metalloproteinase-9 (MMP-9) in serum were quantified using ELISA. Systemic inflammation was evaluated via the CRP/albumin ratio and neutrophil-to-lymphocyte ratio (NLR). Receiver operating characteristic (ROC) curves assessed the diagnostic performance of individual and combined biomarkers, and associations with tumor stage were analyzed according to the AJCC 8th edition.

## Abstract (sr)

**Uvod:**

Planocelularni karcinom arringeala (LSCC) karakterisu složene interakcije između sistemske upale, imunoloske disregulacije i remodeliranja ekstracelularnog matriksa. Cirkulisući biomarkeri koji odražavaju ove bioloske procese mogu pružiti vredne alate za otkrivanje i stratifikaciju bolesti; međutim, njihova integrisana dijagnostička vrednost kod primarnog LSCC ostaje nedovoljno definisana.

**Metode:**

Sproveli smo studiju slučaj-kontrola koja je obuhvatila 50 pacijenata sa LSCC koji nisu primali lečenje i 50 zdravih kontrola. Nivoi YKL-40, rastvorljivog receptora aktivatora plazminogena urokinaze (suPAR) i matriks metalo-proteinaze-9 (MMP-9) u serumu kvantifikovani su pomoću ELISA testa. Sistemska upala je procenjena putem odnosa CRP/albumin i odnosa neutrofila i limfocita (NLR). ROC krive (Receiver Operating Characteristic) procenjivale su dijagnostičke performanse pojedinačnih i kombinovanih biomarkera, a povezanost sa stadijumom tumora analizirana je prema AJCC 8. izdanju.

**Rezultati:**

Pacijenti sa LSCC pokazali su značajno vise serumske nivoe YKL-40 (112,6 ± 28,4 naspram 54,3 ± 15,7 ng/mL), suPAR (4,32 ± 0,98 naspram 2,15 ± 0,61 ng/mL) i MMP-9 (386,5 ± 82,3 naspram 201,7 ± 56,9 ng/mL), zajedno sa povećanim odnosom CRP/Alb (0,186 ± 0,072 naspram 0,062 ± 0,028) i NLR (3,21 ± 1,04 naspram 1,68 ± 0,52) (sve P &lt; 0,001), u poređenju sa zdravim kontrolama. ROC analiza je pokazala dobru dijagnostičku tačnost za pojedinačne biomarkere (AUC vrednosti 0,74 do 0,85), dok je MMP-9 pokazao najveće pojedinačne performanse (AUC = 0,85). Integrisani multivarjantni model koji kombinuj'e YKL-40, suPAR, MMP-9, odnos CRP/Alb i NLR postigao je superiornu dijagnostičku tačnost (AUC = 0,92), sa osetljivošću od 88,0% i specifičnošću od 90,0%.

**Zaključak:**

Integrisani panel biomarkera remodeliranja upale, imunog matriksa i inflamacije je značajno disregulisan kod primarnog LSCC i pokazuje snažnu dijagnostičku i stadijumski stratifikacionu vrednost. Kombinovani model biomarkera nadmasuje pojedinačne markere, sto podržava njegovu korisnost u kliničkoj evaluaciji i proceni rizika.

## Introduction

Laryngeal squamous cell carcinoma (LSCC) is among the most common malignancies of the head and neck and remains a major contributor to cancer-related morbidity worldwide. Despite advances in surgery, radiotherapy, and systemic therapies, early diagnosis and accurate risk stratification are still essential - and challenging - for optimizing patient outcomes [1][2].

Growing evidence indicates that LSCC development and progression are shaped by a dynamic interplay among chronic inflammation, immune dysregulation, and extracellular matrix (ECM) remodeling. Systemic inflammatory responses can promote tumor initiation and support invasion, angiogenesis, and metastatic spread. Accordingly, circulating biomarkers that capture these processes may offer clinically useful information for cancer detection and staging [3][4].

YKL-40 (chitinase-3-like protein 1) is a glycoprotein involved in inflammation, ECM remodeling, and angiogenesis, and elevated levels have been reported across multiple solid tumors [5]. Soluble urokinase plasminogen activator receptor (suPAR) reflects immune activation and proteolytic activity and plays an important role in tumor invasion and metastatic progression [6]. Matrix metalloproteinase-9 (MMP-9), a key mediator of ECM degradation, has been associated with increased tumor aggressiveness and invasive potential [7]. In addition, readily available systemic inflammatory indices derived from routine testing have emerged as practical markers of inflammatory and immune status, with demonstrated prognostic relevance across diverse malignancies [8].

However, few studies have integrated inflammation-, immune-, and matrix remodeling-related biomarkers to assess their combined value for diagnosis and stage stratification in primary LSCC. Therefore, we investigated the clinical utility of YKL-40, suPAR, MMP-9, the CRP/albumin ratio, and the neutrophil-to-lymphocyte ratio (NLR), individually and in combination, for diagnosing and stratifying stage in patients with primary laryngeal cancer.

## Materials and methods

### Study design and participants

This case-control research was carried out between January 2022 and June 2024. A total of 100 participants were recruited and divided into two groups: Primary LSCC group (n = 50): Patients with newly diagnosed, treatment-naïve primary LSCC confirmed by histopathological examination of surgical or biopsy specimens. Healthy control group (n = 50): Age- and sex-matched individuals recruited from routine health examination programs, with no history of malignant disease or chronic inflammatory conditions.

All LSCC cases were staged as per the American Joint Committee on Cancer (AJCC) TNM staging system, 8th edition, based on clinical, imaging, and pathological findings [9].

Inclusion Criteria: Age ≥ 18 years; Histologically confirmed primary LSCC; No prior anti-tumor therapy (surgery, radiotherapy, chemotherapy, or immunotherapy).

Exclusion criteria included: (i) evidenceof recurrent or metastatic laryngeal cancer; (ii) acute infection or inflammatory disease within 4 weeks prior to enrollment; (iii) autoimmune disorders, hematologic diseases, or other chronic inflammatory conditions; (iv) severe hepatic or renal dysfunction; and (v) long-term corticosteroid or immunosuppressive therapy.

The study protocol was approved by the Institutional Ethics Committee of Jieyang People's Hospital, and all participants provided written informed consent. The study was conducted in accordance with the Declaration of Helsinki.

### Blood sample collection and processing

Peripheral venous blood was drawn from all participants between 7:00 and 9:00 a.m. following an overnight fast of at least 8 hours to minimize circadian and dietary influences. Blood intended for serum analysis was collected into serum-separating tubes. The resulting serum was aliquoted and stored at -80°C until laboratory analysis. Blood for complete blood count analysis was collected in EDTA tubes. Repeated freeze-thaw cycles were avoided for all samples.

### Laboratory measurements

### Serum concentrations of YKL-40 (chitinase-3-like protein 1)

Serum YKL-40 concentrations were determined using a commercially available sandwich ELISA kit, following the manufacturer's protocol. The assay exhibited a detection limit below 2.0 ng/mL, and the intra- and inter-assay variability were maintained at less than 8% and 10%, respectively. Each sample was tested in duplicate, and the average of the two measurements was used for subsequent statistical analysis. Calibration curves were constructed using recombinant human YKL-40 standards.

### Serum levels of suPAR

suPAR was quantified via a high-sensitivity ELISA. The assay covered a measurement range of 0.5-10.0 ng/mL, with a minimum detectable concentration of 0.1 ng/mL. Intra-assay and interassay variability were maintained below 7% and 9%, respectively. Optical density readings were obtained at 450 nm using a microplate reader, and sample concentrations were determined based on standard calibration curves.

### Serum MMP-9

Serum MMP-9 concentrations were measured using a quantitative ELISA specifically designed for human MMP-9 with a detection range of 50-1,000 ng/mL and an analytical sensitivity below 10 ng/mL. The intra- and inter-assay variation was maintained below 10%. Samples with concentrations exceeding the upper detection limit were diluted appropriately and re-assayed. Quality control samples with predefined concentrations were included in each analytical batch to ensure assay reliability.

### Serum CRP and albumin levels

Serum CRP and albumin levels were measured following standard laboratory protocols. CRP concentrations were determined by an immunoturbidimetric method, while albumin levels were measured using the bromocresol green colorimetric method. CRP/albumin ratio was calculated to provide a composite index of systemic inflammation and nutritional status.

NLR was measured by dividing absolute neutrophil counts by absolute lymphocyte counts to evaluate systemic immune-inflammatory balance.

### Quality control

All laboratory procedures were performed by experienced technicians blinded to clinical data. Internal quality control samples were included in each analytical run. Duplicate measurements were conducted for all ELISA assays, and any assay with a coefficient of variation exceeding 15% was repeated.

### Statistical analysis

Data analysis was performed using SPSS 26.0. Normality of continuous variables was assessed with the Shapiro-Wilk test. Independent-sample t tests were used for group comparisons between LSCC patients and healthy controls, as well as between tumor stage subgroups. ROC curve analysis was performed to evaluate the diagnostic performance of individual biomarkers and the integrated biomarker model. A multivariate logistic regression model incorporating all biomarkers was used to evaluate combined diagnostic utility. P value <0.05 was considered statistically significant.

## Results

### Baseline clinical characteristics

A total of 100 participants were included in this study, comprising 50 patients with primary LSCC and 50 healthy controls. Among the LSCC patients, tumor staging according to the AJCC 8th edition revealed 28 individuals (56.0%) with early-stage disease (stage I-II) and 22 individuals (44.0%) with advanced-stage disease (stage III-IV) (Table 1).

**Table 1:** Baseline characteristics of LSCC patients and healthy controls.

Variable	LSCC (n = 50)	Controls (n = 50)	P value
Age (years)	59.4 ± 8.7	58.1 ± 9.2	0.48
Male/Female (n)	38 / 12	36 / 14	0.65
Smoking history (%)	64.0	60.0	0.68
Alcohol consumption (%)	46.0	42.0	0.69
Stage I-II (n, %)	28 (56.0)	-	-
Stage III-IV (n, %)	22 (44.0)	-	-

### Comparison of inflammation-immune-matrix remodeling biomarkers between groups

All measured biomarkers were significantly different between LSCC patients and healthy controls. Serum levels of YKL-40, suPAR, and MMP-9 were markedly elevated in the LSCC group, indicating enhanced extracellular matrix remodeling and immune activation. In addition, LSCC patients exhibited significantly higher CRP/albumin ratios and neutrophil-to-lymphocyte ratios (NLR), reflecting a pronounced systemic inflammatory response (Table 2)

**Table 2:** Comparison of biomarker levels between LSCC patients and healthy controls.

Biomarker	LSCC (n = 50)	Controls (n = 50)	P value
YKL-40 (ng/mL)	112.6 ± 28.4	54.3 ± 15.7	<0.001
suPAR (ng/mL)	4.32 ± 0.98	2.15 ± 0.61	<0.001
MMP-9 (ng/mL)	386.5 ± 82.3	201.7 ± 56.9	<0.001
CRP (mg/L)	7.42 ± 3.11	1.86 ± 0.92	<0.001
Albumin (g/L)	39.8 ± 4.6	44.9 ± 3.8	<0.001
CRP/Albumin	0.186 ± 0.072	0.062 ± 0.028	<0.001
NLR	3.21 ± 1.04	1.68 ± 0.52	<0.001

### Diagnostic performance of individual biomarkers

ROC curve analysis was employed to assess the discriminatory performance of each biomarker. Among the individual indicators, MMP-9 achieved the highest diagnostic accuracy (AUC = 0.85), followed by YKL-40 (AUC = 0.83) and suPAR (AUC = 0.80). Inflammatory indices, including the CRP/albumin ratio and NLR, also exhibited acceptable diagnostic performance, though with relatively lower AUC values (Table 3).

**Table 3:** Diagnostic performance of individual biomarkers for LSCC.

Biomarker	AUC	95% CI	Sensitivity (%)	Specificity (%)
YKL-40	0.83	0.75-0.91	78.0	82.0
suPAR	0.80	0.71-0.89	74.0	80.0
MMP-9	0.85	0.77-0.93	82.0	84.0
CRP/Albumin	0.78	0.69-0.87	72.0	76.0
NLR	0.74	0.64-0.84	68.0	72.0

### Diagnostic performance of the integrated biomarker model

**Figure 1:**
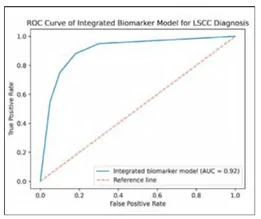
Receiver operating characteristic (ROC) curve of the integrated inflammation-immune-matrix remodeling biomarker model for the diagnosis of primary laryngeal squamous cell carcinoma. The ROC curve illustrates the diagnostic performance of the combined model incorporating YKL-40, soluble urokinase plasminogen activator receptor (suPAR), matrix metalloproteinase-9 (MMP-9), C-reactive protein to albumin ratio (CRP/Alb), and neutrophil-to-lymphocyte ratio (NLR). The integrated model achieved an area under the curve (AUC) of 0.92 (95% CI: 0.86-0.97), demonstrating high sensitivity (88.0%) and specificity (90.0%) for distinguishing LScC patients from healthy controls.

To assess whether combining biomarkers could improve diagnostic accuracy for primary laryngeal squamous cell carcinoma, a multivariate logistic regression model incorporating YKL-40, suPAR, MMP-9, CRP/albumin ratio, and NLR was constructed. ROC analysis revealed that the integrated biomarker model achieved significantly superior diagnostic performance compared with any individual biomarker. As illustrated in Figure 1, the integrated panel achieved an AUC of 0.92 (95% CI: 0.86-0.97), with a sensitivity of 88.0% and specificity of 90.0% for differentiating LSCC patients from healthy individuals. These results indicate that simultaneous assessment of inflammation, immune activation, and extracellular matrix remodeling substantially enhances diagnostic accuracy and provides a more comprehensive biochemical characterization of LSCC.

### Association between biomarkers and tumor stage

Subgroup analysis by tumor stage showed that patients with advanced LSCC (stage III-IV) had significantly higher serum levels of YKL-40, suPAR, and MMP-9 compared with early-stage patients (stage I-II) (P < 0.01). Inflammatory indices also showed an increasing trend with advancing stage, although the differences were less pronounced (Table 4).

**Table 4:** Comparison of biomarker levels according to tumor stage in LSCC patients.

Biomarker	Stage I-II (n = 28)	Stage III-IV (n = 22)	P value
YKL-40 (ng/mL)	98.4 ± 21.6	130.2 ± 24.9	<0.001
suPAR (ng/mL)	3.86 ± 0.72	4.91 ± 0.88	<0.001
MMP-9 (ng/mL)	345.7 ± 68.4	438.9 ± 74.2	<0.001
CRP/Albumin	0.162 ± 0.058	0.217 ± 0.081	0.008
NLR	2.89 ± 0.83	3.62 ± 1.14	0.012

## Discussion

This study comprehensively evaluated the diagnostic and stage-stratification value of an integrated panel of inflammation-immune-matrix remodeling biomarkers in patients with primary LSCC. The main findings can be summarized as follows: (1) serum YKL-40, suPAR, MMP-9, the CRP/albumin ratio, and NLR were significantly higher in LSCC patients than in healthy controls; (2) although each biomarker showed acceptable diagnostic performance when assessed individually, the combined panel achieved superior discriminative accuracy; and (3) higher levels of YKL-40, suPAR, and MMP-9 were closely associated with advanced tumor stage. Taken together, these results support the notion that LSCC involves coordinated dysregulation of systemic inflammation, immune activation, and extracellular matrix remodeling.

Chronic inflammation is a well-established hallmark of carcinogenesis, including in head and neck malignancies [10][11]. In this study, the elevated CRP/albumin ratio and NLR in LSCC patients indicate a systemic inflammatory response accompanied by immune imbalance. Increased CRP reflects acute-phase activation, whereas lower albumin may result from inflammation-related shifts in hepatic protein synthesis and cancer-associated nutritional impairment. Accordingly, the CRP/albumin ratio provides an integrated measure of inflammatory burden and metabolic stress, which was markedly higher in LSCC patients in the present cohort.

NLR provides further insight into the balance between pro-tumor inflammatory activity and anti-tumor immune surveillance [12][13]. Neutrophils can promote tumor growth through the release of reactive oxygen species, proteases, and proangiogenic factors, while lymphopenia may reflect impaired cellular immune responses [14]. The significantly elevated NLR in LSCC patients observed here is consistent with previous evidence linking immune dysregulation to tumor progression.

Beyond systemic inflammation, tumor-driven extracellular matrix (ECM) remodeling is pivotal to invasion and metastasis. MMP-9, a protease that degrades type IV collagen, facilitates basement membrane disruption and promotes tumor cell invasion and metastatic dissemination [15]. In the present study, serum MMP-9 levels were markedly elevated in LSCC patients, particularly in those with advanced-stage disease, supporting its association with aggressive tumor behavior.

YKL-40 and suPAR are key mediators at the interface of inflammation, immune activation, and tissue remodeling [16]. YKL-40 has been linked to angiogenesis, fibrotic remodeling, and tumor progression [17]. Its significantly increased serum levels in LSCC patients, together with the strong association with tumor stage, suggest that YKL-40 may reflect both tumor-related stromal activity and concomitant systemic inflammatory responses.

Similarly, suPAR reflects activation of the plasminogen activation system [18][19]. Elevated suPAR levels indicate enhanced proteolysis, immune activation, and cell migration - processes that are essential for tumor invasion and metastasis [20]. The robust diagnostic performance of suPAR observed in this study supports its potential role as a circulating marker of tumor aggressiveness in LSCC.

Importantly, the combined biomarker model integrating YKL-40, suPAR, MMP-9, the CRP/ albumin ratio, and NLR significantly outperformed any single marker in discriminating LSCC patients from healthy controls. Whereas individual biomarkers capture specific biological processes, carcinogenesis is multifactorial and systemic. Accordingly, integrating markers that represent complementary pathways - systemic inflammation, immune activation, and extracellular matrix remodeling - may offer a more comprehensive biochemical profile of LSCC and thereby improve diagnostic characterization.

The integrated panel achieved a high diagnostic accuracy (AUC = 0.92), demonstrating its potential as a non-invasive tool for early detection and risk stratification in primary LSCC. Importantly, all components of this biomarker panel are measurable using routine laboratory techniques, which enhances its translational feasibility.

The progressive increase in YKL-40, suPAR, and MMP-9 levels with advancing tumor stage further supports their biological relevance to LSCC progression. These findings suggest that tumor burden and invasiveness are closely linked to systemic inflammatory activation and extracellular matrix degradation. Although inflammatory indices such as CRP/albumin ratio and NLR also showed stage-related trends, their associations were less pronounced, possibly reflecting their broader sensitivity to non-tumor-related inflammatory stimuli.

Taken together, these results indicate that inflammation-immune-matrix remodeling biomarkers may provide clinically valuable information for both diagnosis and stage stratification.

Limitations of this study should be acknowledged. First, the sample size was relatively small, and the study was conducted at a single center, which may limit generalizability. Second, this study focused on diagnostic and stage stratification value, without evaluating long-term prognostic outcomes such as survival or treatment response. Third, although confounding inflammatory conditions were carefully excluded, subclinical inflammatory states cannot be completely ruled out. Future multicenter studies with larger cohorts and longitudinal follow-up are warranted to validate and extend these findings.

## Conclusion

In conclusion, this study demonstrates that an integrated panel of inflammation-immune-matrix remodeling biomarkers, including YKL-40, suPAR, MMP-9, CRP/albumin ratio, and NLR, is significantly dysregulated in patients with primary laryngeal squamous cell carcinoma. The combined biomarker model provides superior diagnostic accuracy compared with individual markers and effectively discriminates between early- and advanced-stage disease. These findings highlight the potential clinical value of integrated biochemical biomarkers as non-invasive tools for diagnosis and stage stratification in laryngeal cancer, and support further investigation into their role in personalized cancer management.

## Dodatak

### Conflict of interest statement

All the authors declare that they have no conflict of interest in this work.
